# Exploring the Dynamics of Artificial Intelligence Literacy on English as a Foreign Language Learners’ Willingness to Communicate: The Critical Mediating Roles of Artificial Intelligence Learning Self-Efficacy and Classroom Anxiety

**DOI:** 10.3390/bs15040523

**Published:** 2025-04-14

**Authors:** Qinqing Zhang, Hua Nie, Jiqun Fan, Honggang Liu

**Affiliations:** 1 School of Foreign Languages, Huainan Normal University, Huainan 232001, China; zhangqinqing@hnnu.edu.cn (Q.Z.); 106050@hnnu.edu.cn (J.F.); 2 School of Foreign Studies, China University of Political Science and Law, Beijing 100088, China; 3 School of Foreign Studies, Soochow University, Suzhou 215006, China

**Keywords:** artificial intelligence (AI), AI literacy, willingness to communicate, AI learning self-efficacy, foreign language classroom anxiety

## Abstract

The increasing incorporation of artificial intelligence (AI) in English as a foreign language (EFL) instruction has garnered much attention on the importance of technological elements in language instruction. However, while AI in education (AIED) is still in its early development, research on how learners’ AI literacy affects their language learning outcomes is insufficient. Furthermore, studies examining the impact of learners’ emotional states within the context of AIED are remarkably few. This study examines the interplay between AI literacy and EFL learners’ willingness to communicate (WTC), emphasizing the mediating roles of learners’ AI learning self-efficacy and foreign language classroom anxiety. This study utilizes structural equation modeling, analyzing data from 517 university students in China to construct a prediction model for WTC in AI-enhanced EFL contexts. The findings indicate that AI literacy improves self-efficacy in AI learning and diminishes classroom anxiety, both of which are significant mediators in the relationship between AI literacy and willingness to communicate. The study highlights the imperative of integrating AI literacy into EFL instruction to enhance learners’ expressive confidence and mitigate fear. The findings improve understanding of the interplay between AI literacy, psychological factors, and language learning outcomes, offering practical insights for the integration of AI in EFL education.

## 1. Introduction

The breadth and depth of technology-enhanced English as a foreign language (EFL) instruction have increased in recent decades due to the quick development of computer and Internet technologies. The incorporation of contemporary educational technology in EFL teaching has elevated the technological proficiency demanded from both educators and learners. Research has identified various literacies—information literacy, computer literacy, ICT literacy, media literacy, digital literacy, and AI literacy—each emphasizing specific facets of learners’ technological competence within the context of technology-enhanced EFL instruction (Hernández-Marín et al., 2024; Nichols & Stornaiuolo, 2019; H. Park et al., 2021; Zhang et al., 2024). The paramount importance of technology in EFL research and education is increasingly acknowledged (Lee, 2019; Lee & Hsieh, 2019; Reinders & Wattana, 2015; Zare et al., 2022).

In conventional EFL instruction, particularly in listening and speaking, willingness to communicate (WTC) has been a prominent topic of scholarly investigation. Empirical research conducted in various global locations over the past five years demonstrates that EFL learners’ WTC is influenced by numerous factors, including the learning environment, intrinsic motivation, self-efficacy, emotional states, and personal backgrounds (Minguez-Alcaide & Perez-Tejada, 2023; Y. L. Wang et al., 2024). The thorough integration of modern educational technology, especially computer-assisted tools, into EFL instruction has garnered significant attention from scholars about the relationship between technological settings and learners’ communicative objectives (Hsieh et al., 2023; H. Huang & Li, 2024). Over the past decade, the advent of artificial intelligence in education (AIED) has emerged as a trend in EFL instruction, addressing learners individualized educational needs and promoting their autonomy and independence (Cacho, 2024; Y. Park & Doo, 2024). The impact of AI literacy, defined as EFL learners’ ability to understand and effectively employ AI technologies, on learners’ WTC has not been extensively examined.

Conventional educational frameworks in EFL research have recognized individual learner emotions and psychological attributes, such as self-efficacy, enjoyment, resilience, and anxiety, as important factors influencing WTC (Al-Saidat et al., 2023; Lee & Chiu, 2023). Nevertheless, within an AIED framework, the influence of technological factors, such as learners’ AI literacy, on their emotional states and the subsequent effect on WTC remains little examined and lacks empirical evidence.

This research, based on self-efficacy theory, formulates a predictive model for EFL learners’ WTC inside an AIED framework. It investigates the correlation among learners’ AI literacy, self-efficacy, and anxiety within foreign language classrooms, as well as the impact of individual psychological emotions on AI-enhanced EFL education. This research intends to enhance our understanding of the impact of technological factors on WTC in AI-enhanced EFL learning and to clarify the importance of learners’ particular psychological states. The study’s conclusions hold empirical significance, highlighting the crucial role of learners in AIED environments.

## 2. Literature Review

### 2.1. Willingness to Communicate

Willingness to communicate refers to an individual’s readiness to enter into conversation with specific people under certain conditions using a language (Baker & Watson, 2015). It is considered a critical component in language learning and communication contexts, as it reflects a person’s intention to initiate and engage in discourses (Zare et al., 2022). Initially, it was developed to account for individual differences in first language communication (McCroskey & Richmond, 1990). Over time, it has been expanded to explain second language learning and communication, particularly in non-English-speaking countries where English is often taught as a foreign language (Baker & MacIntyre, 2000; M. Liu, 2017). Previous research has identified that the willingness of learners to engage in communicative language activities is primarily influenced by sociocultural and individual psychological factors. These factors are predominantly manifested in aspects such as individual cultural background, cognitive abilities, teacher–student interaction, and learners’ personal emotions (Jelinková et al., 2023; Pawlak & Mystkowska-Wiertelak, 2015; Weda et al., 2021). Despite the extensive research on WTC in the context of EFL instruction, studies examining WTC within the context of AIED remain relatively limited, especially in the era where AI technologies are increasingly integrated into EFL education (Fathi et al., 2024; F. Huang & Zou, 2024).

### 2.2. AI Literacy

AI literacy (AIL), as delineated in contemporary scholarly discourse, refers to an individual’s capacity to harness AI-related knowledge and skills, thereby fostering a robust comprehension of AI’s foundational principles and practical applications (Belda-Medina & Calvo-Ferrer, 2022; Dai et al., 2020). Within the educational sphere, the integration of AI literacy into curricula is essential for equipping students to navigate an AI-augmented future. This preparation entails the cultivation of a comprehensive understanding of AI, encompassing its underlying mechanisms, the critical evaluation of its societal implications, and the development of competencies for effective collaboration with AI systems (Allen & Kendeou, 2024). In the realm of EFL education, AIL is becoming crucial for learners, enabling them to critically understand and assess AI-driven applications in educational platforms and tools. Nonetheless, the relationship between EFL learners’ AI literacy and their language learning outcomes has received limited attention in current research, with even less exploration of the pathways through which this relationship operates.

### 2.3. Foreign Language Classroom Anxiety

The emotional landscape of foreign language learning has long intrigued second language acquisition scholars, with initial research predominantly focusing on the detrimental role of negative emotions in the language learning process. MacIntyre (1999) conceptualizes foreign language classroom anxiety (FLCA) as “the worry and negative emotional reaction aroused when learning or using a second language,” which is shaped by the specific challenges and experiences inherent to learning a language within an educational setting (Dewaele et al., 2024).

FLCA is intrinsically linked to feelings of apprehension, trepidation, and nervousness that learners often encounter during the process of acquiring a foreign language. It fosters a sense of insecurity and the perception of psychological threats, potentially stifling linguistic development and classroom participation (Alberth, 2022). This constellation of negative emotions can significantly impede the optimal performance of language learners, particularly their willingness to communicate (Amirian et al., 2022; Baran-Lucarz, 2014; Bensalem, 2022; Dewaele, 2019).

With the widespread application of AI in EFL education, the impact of AI technology on learners’ FLCA and the influence of FLCA on EFL learning outcomes in the context of AI in AIED warrant further exploration.

### 2.4. Self-Efficacy Theory and AI Learning Self-Efficacy

As a fundamental concept within educational theory, self-efficacy, initially conceptualized by Bandura (Bandura & Wessels, 1997) as part of social cognitive theory, refers to an individual’s fundamental belief in their capabilities to navigate and triumph over difficult situations. This overarching belief system further differentiates into task-specific self-efficacy, which provides a more detailed assessment of an individual’s confidence in their ability to excel in specific tasks or environments (Q. Liu et al., 2020). Self-efficacy is influenced by a range of factors, including mastery experiences, vicarious experiences, social persuasion, psychological and physiological states, goal setting, social support, individual differences, and educational environment (Calaguas & Consunji, 2022; D. Shen et al., 2013; Yesilyurt et al., 2016). The self-efficacy theory posits that individuals’ beliefs in their capabilities to perform specific tasks significantly influence their motivation, behavior, and performance outcomes (H. Liu et al., 2024).

In the domain of AI, the concept of self-efficacy has been specialized to address a particular facet: AI learning self-efficacy (ALSE). This construct differs from the broader notion of self-efficacy by focusing on an individual’s confidence in their ability to perform specific tasks or navigate particular situations that are centered around AI technologies. ALSE evaluates how well-equipped and assured individuals feel when it comes to applying AI tools and systems effectively to achieve their goals (Y.-Y. Wang & Chuang, 2024). This tailored measure of self-efficacy is crucial as AI becomes more integrated into various fields, influencing not only the performance but also the learning and adoption of AI technologies (D. Chen et al., 2024). Nevertheless, the empirical investigation of self-efficacy’s influence on the acquisition of novel AI technologies remains scarce (Y.-M. Wang et al., 2024).

### 2.5. The Present Study and Hypotheses

**Figure 1:**
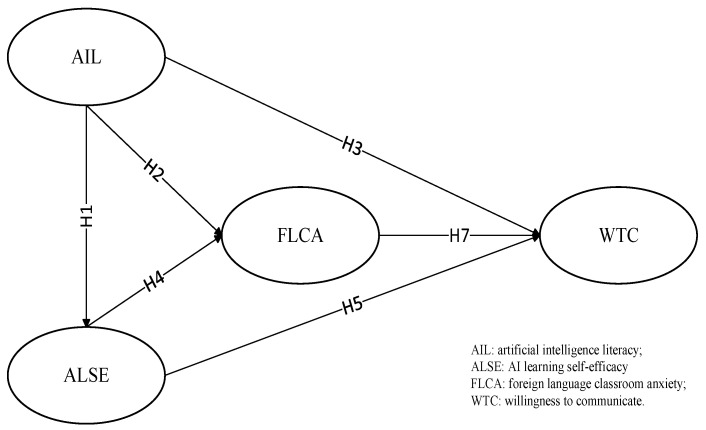
Hypothesized model.

This study, guided by self-efficacy theory, explores the dynamic relationships among learners AIL, AISE, FLCA, and WTC in AI-assisted EFL teaching contexts (see Figure 1).

Literacy, as an indicator of individual cognitive ability, has been shown to positively predict self-efficacy (Niu et al., 2021; C. Park, 2024). This relationship is particularly pronounced in the context of digital literacy. For instance, Gumus et al. (2024) found that digital literacy significantly enhances secondary school students’ self-efficacy in both computer programming and computational thinking. Similarly, Dai (2024) demonstrated that AI literacy among upper elementary students (ages 10–12) can substantially mitigate classroom tension. Although the existing literature offers limited evidence regarding the predictive role of AI literacy on communicative willingness, X. Shen et al. (2024) empirically confirmed that digital literacy positively influences the communicative willingness of second language learners. Building on these findings, the following hypotheses are proposed for this study:

**H1.**  *EFL learners’ AI literacy positively influences their AI learning self-efficacy.*

**H2.**  *EFL learners’ AI literacy negatively influences their foreign language classroom anxiety.*

**H3.**  *EFL learners’ AI literacy positively influences their willingness to communicate.*

Learners’ self-efficacy reflects their confidence in their own learning behaviors. In the context of AI in education (AIED), D. Chen et al. (2024) found that second language learners’ AI self-efficacy negatively impacts their anxiety, which in turn affects their use of AI tools. Similarly, Li et al. (2024) empirically demonstrated in their study on Chinese learners’ willingness to use ChatGPT-3.5 that AI learning self-efficacy positively predicts communicative willingness. Furthermore, the mediating role of self-efficacy in AIED contexts has been confirmed in prior research (B.-J. Kim & Kim, 2024; B.-J. Kim & Lee, 2024). Based on the above analysis, the following hypotheses are proposed for this study:

**H4.**  *EFL learners’ AI learning self-efficacy negatively influences their foreign language classroom anxiety.*

**H5.**  *EFL learners’ AI learning self-efficacy positively influences their willingness to communicate.*

**H6.**  *EFL learners’ AI learning self-efficacy acts as a mediator between EFL learners’ AI literacy and their willingness to communicate.*

Prior research has conclusively demonstrated that foreign language classroom anxiety significantly hampers learners’ willingness to communicate (Amirian et al., 2022; Dewaele, 2019). This adverse relationship has also been empirically confirmed in the context of AI in education, where studies have shown that learner anxiety negatively predicts foreign language communicative willingness (H. Liu & Fan, 2025; Zhang et al., 2024). Additionally, the mediating role of anxiety has been extensively examined in prior studies. Based on these findings, the following research hypotheses are proposed in this study:

**H7.**  *EFL learners’ foreign language classroom anxiety negatively influences their willingness to communicate.*

**H8.**  *EFL learners’ foreign language classroom anxiety acts as a mediator between EFL learners’ AI literacy and their willingness to communicate.*

**H9.**  *EFL learners’ AI learning self-efficacy and their foreign language classroom anxiety sequentially mediate the relationship between EFL learners’ AI literacy and their willingness to communicate.*

## 3. Method

### 3.1. Participants and Procedure

The study population consisted of 517 non-English major undergraduates from four higher educational institutions in Anhui Province, China. Participants were enrolled in university English-speaking courses that employed artificial intelligence (AI)-assisted software. Teachers encouraged students to utilize AI software across various terminal devices, including smartphones and desktop computers. In the domain of English language acquisition, participants frequently employed AI technology for voice correction training, vocabulary retention, automatic translation, corpus development, information retrieval, speaking practice with AI companions, and voice transcription. The respondents consisted of 318 females and 199 males. The demographic data are provided in Table 1.

The survey employed random sampling, focusing on the communicative willingness of learners in AI-augmented EFL learning contexts. Questionnaires were distributed by EFL instructors utilizing AI technology in their various classrooms. Before the release of the questionnaires, participants were briefed on the study’s objectives and guaranteed the confidentiality of their information. They were directed to respond to the questions with accuracy but were also notified that they might withdraw at any moment if they chose not to participate. Participants received a canvas tote bag as a token of appreciation upon completing the survey. Over a three-week duration, 650 questionnaires were distributed, yielding 588 returns and 517 valid responses.

### 3.2. Instruments

To construct a model for predicting the willingness to communicate in foreign languages among EFL learners in an AIED context and to explore the interactive relationships between learners’ artificial intelligence literacy, self-efficacy in AI learning, foreign language classroom anxiety, and willingness to communicate in foreign languages, this research conducted a survey on the subjects using relevant questionnaires. The participants’ replies on the relevant problems are assessed using a 5-point Likert scale, where 1 signifies “strongly disagree” and 5 denotes “strongly agree”.

The study utilized Chai et al. (2020)’s AIL scale, which assesses the degree to which EFL learners comprehend the operational mechanisms and cognitive processes underlying AI-driven learning technologies. The scale encompasses six items, including “I know the processes through which deep learning enables AI to perform voice recognition tasks”. In this study, a Cronbach’s α coefficient of 0.914 was reported for AIL, signifying substantial internal consistency.

The assessment of AI learning self-efficacy utilized the scale created by Y.-M. Wang et al. (2024), comprising five items (e.g., “I do not think I lack the foundation for AI-related skills learning.”). The scale is designed to measure learners’ self-efficacy in learning AI technologies, which aligns with the context established for this study. In the present study, the reliability index of ALSE estimated through Cronbach’s alpha was 0.871, signifying adequate internal consistency.

The current research employed the instrument created by Dewaele and MacIntyre (2014) to evaluate participants’ perceptions of anxiety in foreign language educational settings. The scale in question is a classic instrument for measuring foreign language learners’ classroom anxiety and has been widely employed by numerous scholars across different educational settings. This scale comprises eight items (e.g., “I get nervous and confused when I am speaking in my foreign language class”). The analysis revealed FLCA enjoyed good internal consistency (Cronbach’s α = 0.942).

This study assessed EFL learners’ willingness to communicate using a modified scale by Dewaele (2019). Since its introduction, the scale has been widely employed to assess foreign language learners’ willingness to communicate. It consists of six items (e.g., “I can imagine a situation where I am speaking English with foreigners.”). In the current study, the reliability of learners’ WTC scale estimated via Cronbach’s α was 0.946, indicating substantial internal consistency.

### 3.3. Data Analysis

The data were analyzed utilizing SPSS version 26.0 and AMOS version 24.0. Initial descriptive and correlational analyses were performed to summarize the data. Subsequently, confirmatory factor analysis was utilized to validate the measurement model. Subsequently, structural equation modeling was employed to assess the model’s fit and to investigate the correlations via regression and mediation analysis. The goodness-of-fit indices conformed to the following criteria: χ^2^/df < 3, RMSEA ≤ 0.08, SRMR ≤ 0.10, GFI, CFI, TLI, and AGFI > 0.90 (Kline, 2023).

## 4. Results

### 4.1. Test for Common Method Variance

To investigate common method bias, the present study employs the Harman single-factor test (Podsakoff et al., 2003). All four factors were included in the maximum likelihood extraction method. The value of Harman’s single factor is 32.135%, well below the critical threshold of 40% (Hair et al., 1998). The common method bias was not a significant issue in the present study.

### 4.2. Measurement Analysis

This study utilized SPSS 26.0 to evaluate the measurement model’s reliability and validity. The eigenvalue method detected four prominent constructs, each with a loading beyond 0.65. The internal consistency among items within each dimension is robust, since the Cronbach’s alpha for each construct was above 0.7. Furthermore, as Table 2 indicated, the composite reliability (CR) and average variance extracted (AVE) of each dimension were higher than the threshold criteria of 0.7 and 0.5, respectively. The square root values of AVE for each construct surpassed the correlation between these constructs (refer to Table 3), meeting the requirement of the discriminant validity.

### 4.3. Test for Structural Model

**Figure 2:**
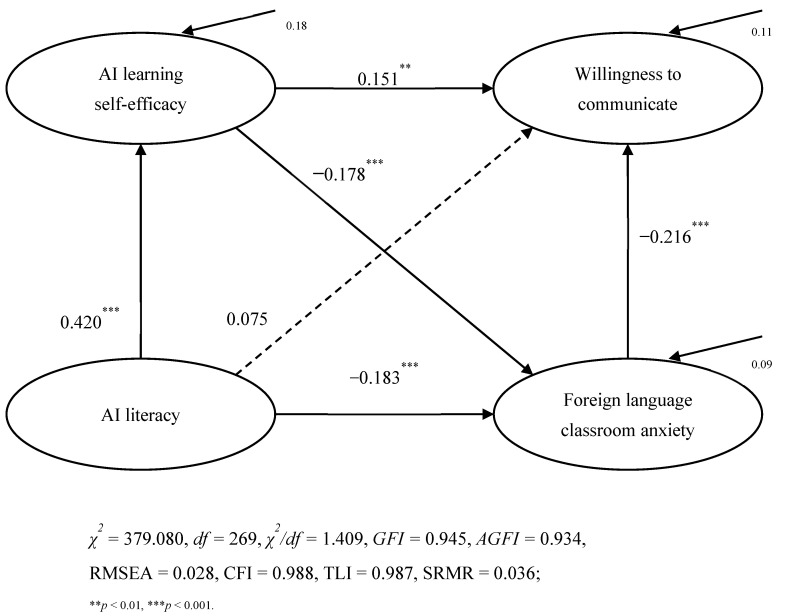
SEM test model.

This study utilized AMOS 24.0 to do a goodness-of-fit examination of the theoretical research model. According to Kline’s guidelines, the optimal model fit indices are χ^2^/df < 3, with CFI, GFI, IFI, AGFI, and TLI ≥ 0.90, and RMSEA and SRMR ≤ 0.08. In this study, χ^2^ equals 379.080 with degrees of freedom (df) equal to 269, yielding a χ^2^/df ratio of 1.409. The other fit indices, including GFI (0.945), AGFI (0.934), CFI (0.988), TLI (0.987), RMSEA (0.028), and SRMR (0.036), satisfied the requirements (see Figure 2). The results demonstrate strong data and model alignment, facilitating additional empirical investigation.

Table 4 illustrates that in an AI-augmented EFL learning context, learners’ artificial intelligence literacy positively impacts their self-efficacy on AI learning (β = 0.420, *p* < 0.001, SE = 0.033) and negatively predicts their foreign language classroom anxiety (β = −0.183, *p* < 0.001, SE = 0.041), hence corroborating hypotheses H1 and H2. However, the study revealed that learners’ AI literacy did not substantially affect their preparedness to communicate (β = 0.075, *p* = 0.147, SE = 0.041), leading to the dismissal of hypothesis H3. Concurrently, learners’ self-efficacy in AI learning negatively predicted their anxiety in foreign language classrooms and positively influenced their communication preparedness (β = −0.178, *p* < 0.001, SE = 0.062; β = 0.151, *p* = 0.004, SE = 0.063), thereby substantiating hypotheses H4 and H5. The results indicated that learners’ anxiety in foreign language classrooms negatively impacted their readiness to speak (β = −0.216, *p* < 0.001, SE = 0.049), hence supporting hypothesis H7.

### 4.4. Mediation Analysis

The findings indicate an insignificant direct effect of AIL on WTC in the proposed model (*p* = 0.161), suggesting full mediation. The results show that AIL has a significant indirect effect on WTC through both ALSE (β = 0.051, SE = 0.020, 90% CI [0.015, 0.093]) and FLCA (β = 0.032, SE = 0.014, 90% CI [0.010, 0.065]), thereby supporting the establishment of hypotheses H6 and H8. Moreover, the study demonstrates that ALSE and FLCA together exert a serial mediating effect on the relationship between AIL and WTC (β = 0.013, SE = 0.005, 90% CI [0.005, 0.025]), confirming hypothesis H9. Among the three mediating pathways, the sequence “AIL → ALSE → WTC” accounts for the highest proportion of the indirect effect, at 53.2% (see Table 5).

## 5. Discussion

The study employed variables such as EFL undergraduates’ AI literacy, foreign language classroom anxiety, and AI learning self-efficacy to develop a predictive model for their willingness to communicate in an AI-enhanced educational environment.

Firstly, the finding that EFL learners’ AIL positively influences their ALSE aligns with the work of Yao and Wang (2024), who demonstrated that digital literacy significantly impacts self-efficacy in the context of AIED for preservice special-education teachers. This consistency underscores the pivotal role of technological proficiency in bolstering learners’ confidence when utilizing AI tools for educational purposes. In the current study, this positive effect is attributed to the empowering nature of AI tools, which provide learners with greater control over their learning process and offer immediate, personalized feedback. This enhanced control and feedback mechanism reinforces learners’ belief in their capacity to effectively employ AI for language learning, thereby augmenting their self-efficacy. Moreover, the inverse relationship between AIL and FLCA is corroborated by D. Chen et al. (2024), who found that higher AI learning self-efficacy among Chinese college students learning a second language was associated with lower AI anxiety and more positive perceptions of AI technology utilization. In the current study, this negative correlation can be explained by the fact that increased AI literacy empowers learners with a better understanding and control over AI tools, which in turn reduces their anxiety levels. As learners become more proficient in using AI for language learning, they feel more confident and less anxious about engaging with these technologies in the classroom setting.

Secondly, the finding that EFL learners’ ALSE negatively impacts their FLCA is consistent with previous research showing a negative correlation between self-efficacy and anxiety in various educational contexts (Awofala et al., 2019; Chou, 2001; Kutuk et al., 2022; X. Wang et al., 2023). This suggests that higher ALSE can reduce FLCA. In AI-enhanced EFL settings, AI tools provide immediate feedback and personalized learning, which can boost learners’ confidence and reduce anxiety. Thus, fostering AI literacy and self-efficacy can create a more positive learning environment. The present study also indicated that AIL does not directly predict WTC. This contrasts with X. Shen et al. (2024), who empirically demonstrated a positive impact of digital literacy on communicative willingness among second language learners. The discrepancy likely arises because AIL, while boosting technical proficiency, does not directly address the psychological and emotional factors influencing WTC. Essentially, WTC is driven by subjective psychological emotions rather than solely by learners’ basic cognitive abilities. Thus, AIL alone cannot significantly impact WTC without mediating factors addressing these psychological aspects.

Finally, the study found that learners’ self-efficacy in AI learning mediates the relationship between AI literacy and their readiness to communicate in English, echoing Ibrahim and Aldawsari (2023), who showed that self-efficacy affects the link between digital competence and academic performance. This indicates that self-efficacy is key in translating AI literacy into communicative readiness. Confidence in using AI for language learning enhances learners’ willingness to communicate, highlighting the need to boost both technical skills and self-efficacy. The study also reveals that in AI contexts, learners’ FLCA significantly mediates the relationship between their AIL and WTC. This finding is consistent with previous research indicating that anxiety acts as a strong moderator influencing learners’ readiness to interact in both online and offline settings in non-AI contexts (Brauer et al., 2023; Hejazi et al., 2023; Piechurska-Kuciel et al., 2021). The mediating role of anxiety in the current study suggests that even with enhanced AI literacy, learners’ WTC is still heavily influenced by their emotional state. High levels of anxiety can inhibit the positive effects of AI literacy on communication, highlighting the importance of addressing emotional barriers alongside technological proficiency in AI-enhanced language learning environments. Furthermore, the study finds that ALSE and FLCA successively mediate the relationship between AIL and WTC. This aligns with prior EFL research highlighting self-efficacy and anxiety as key mediators in language learning (Jin et al., 2024; X. Wang et al., 2023). In AI-enhanced EFL contexts, higher AIL boosts ALSE, as learners gain confidence in using AI tools. This increased self-efficacy then reduces FLCA, making the learning environment less intimidating. Thus, addressing both psychological factors (self-efficacy and anxiety) alongside AIL is crucial for enhancing communicative competence.

## 6. Conclusions

This study investigated Chinese non-English major university students and used structural equation modeling to analyze the interplay between AI literacy, AI learning self-efficacy, and foreign language classroom anxiety in AI-enhanced EFL environments. It affirmed that AI literacy improves self-efficacy and diminishes classroom anxiety, hence affecting communication willingness—positively through self-efficacy and negatively through anxiety. The study revealed that AI literacy does not significantly influence communicative willingness, suggesting that self-efficacy and classroom anxiety completely mediate this relationship in AI-supported EFL educational environments.

This study contributes theoretically by elucidating the intricate interplay between AI literacy, psychological factors (self-efficacy and anxiety), and communicative willingness in AI-enhanced EFL contexts, and practically by offering insights for integrating AI literacy into EFL education to optimize learning outcomes and mitigate anxiety. To begin with, universities must align with the AI education landscape by leveraging the positive role of AI technology in foreign language learning while recognizing that mere literacy education has limited direct influence on learners’ communicative willingness. In response, higher education institutions can embed AI foundational theory and practical application modules into existing foreign language courses (X. Liu & Xiao, 2025; Namaziandost & Rezai, 2024), helping students understand AI’s role in language learning and emphasizing the importance of enhancing personal AI literacy during language training.

Next, educators should harness the impact of AI technology on foreign language teaching by using AI tools (e.g., chatbots) to support classroom interaction and group discussions (Jiang, 2025; Kong et al., 2025). They can employ human–AI interactive games to reduce classroom anxiety and utilize intelligent learning companions to boost students’ AI learning self-efficacy and adaptive learning abilities, enabling learners to more effectively express their viewpoints in a foreign language (M.-R. A. Chen, 2024; Xin & Derakhshan, 2025; Zhang et al., 2024).

Lastly, software developers should prioritize enhancing the interactivity of AI software to lower the learning threshold and establish good user habits (Ng et al., 2024). AI software should provide emotional support to help students overcome psychological barriers such as anxiety and lack of confidence, offering positive feedback and encouragement during human–AI interactions to enhance learners’ self-efficacy and improve their willingness and skills to communicate (F. Huang et al., 2024; Ishizu et al., 2024).

The study provides insights into the relationship between AIL and WTC among EFL learners but has notable limitations. First, the sample of Chinese non-English major undergraduates limits the generalizability of the findings to other regions or educational contexts. Future research should include more diverse samples to enhance applicability. Second, the cross-sectional design and self-reported data capture attitudes at a single point in time but do not track changes in psychological states or communicative goals over time. Longitudinal studies using latent growth models could provide a more nuanced understanding of these dynamics. Third, self-reported data may be biased by social desirability, potentially affecting accuracy. Future research should integrate qualitative methods, such as interviews and classroom observations, to validate findings and offer deeper insights into learners’ experiences.

## Figures and Tables

**Table 1:** Individual’s demographic characteristics.

Category	N	%
Gender		
Female	318	61.5
Male	199	38.5
Grade		
Freshmen	102	19.7
Sophomore	89	17.2
Junior	166	32.1
Senior	60	11.6
Age		
≤19	105	20.3
20	144	27.9
21	195	37.7
≥22	72	14.1
Field of study		
Natural science	110	21.3
Engineering and technical study	172	33.2
Humanities	235	45.5
L (Length of weekly AI use)		
L ≤ 1 h	95	18.40%
1 h < L ≤ 3 h	190	36.8
3 h < L ≤ 7 h	117	22.6
7 h < L ≤ 10 h	56	10.8
10 h < L ≤ 15 h	12	2.3
L > 15 h	47	9.1
Total	517	

**Table 2:** Factor analysis, construct reliability and convergent validity.

Constructs	Items	Estimate	S.E.	Z Value	*p*	Factor Loading	α	C.R.	AVE
FLCA	FLCA01	1.000			***	0.854	0.942	0.942	0.671
	FLCA02	0.889	0.038	23.462	***	0.817			
	FLCA03	0.878	0.037	23.671	***	0.821			
	FLCA04	0.909	0.040	22.823	***	0.804			
	FLCA05	0.848	0.036	23.475	***	0.817			
	FLCA06	0.935	0.035	26.661	***	0.878			
	FLCA07	0.853	0.038	22.401	***	0.794			
	FLCA08	0.829	0.040	20.989	***	0.763			
AIL	AIL1	1.000			***	0.816	0.914	0.923	0.667
	AIL2	0.981	0.049	20.277	***	0.781			
	AIL3	0.979	0.049	19.869	***	0.778			
	AIL4	1.021	0.047	21.535	***	0.825			
	AIL5	0.931	0.046	20.171	***	0.787			
	AIL6	0.988	0.047	20.855	***	0.906			
ALSE	ALSE1	1.000			***	0.798	0.871	0.873	0.580
	ALSE2	0.997	0.049	20.277	***	0.842			
	ALSE3	0.886	0.050	17.597	***	0.744			
	ALSE4	0.886	0.051	17.475	***	0.740			
	ALSE5	0.759	0.048	15.664	***	0.674			
WTC	WTC1	1.000			***	0.883	0.946	0.947	0.750
	WTC2	1.030	0.034	30.566	***	0.904			
	WTC3	0.844	0.031	27.212	***	0.857			
	WTC4	0.964	0.034	28.771	***	0.880			
	WTC5	0.897	0.033	27.333	***	0.859			
	WTC6	0.734	0.030	24.396	***	0.810			

Note: *** *p* < 0.001.

**Table 3:** Correlation matrices and discriminant validity.

Constructs	AIL	ALSE	FLCA	WTC
AIL	0.817			
ALSE	0.42	0.762		
FLCA	−0.258	−0.255	0.819	
WTC	0.194	0.237	−0.274	0.866

Note: AIL denotes artificial intelligence literacy, ALSE signifies AI learning self-efficacy, FLCA represents foreign language classroom anxiety, and WTC indicates willingness to communicate.

**Table 4:** Path analyses.

Hypothesis	Path	B	SE	Z	*p*	β	Hypotheses
H1	AIL → ALSE	0.281	0.033	8.553	***	0.420	Supported
H2	AIL → FLCA	−0.144	0.041	−3.529	***	−0.183	Supported
H3	AIL → WTC	0.06	0.041	1.451	0.147	0.075	Rejected
H4	ALSE → FLCA	−0.209	0.062	−3.372	***	−0.178	Supported
H5	ALSE → WTC	0.181	0.063	2.867	0.004	0.151	Supported
H7	FLCA → WTC	−0.222	0.049	−4.54	***	−0.216	Supported

Note: *** *p* < 0.001.

**Table 5:** Mediation Analysis.

Path	β			95% CI		
		SE	Z-Value	LL	UL	*p*
AIL → ALSE → FLCA → WTC	0.013	0.005	2.600	0.005	0.025	0.001
AIL → ALSE → WTC	0.051	0.020	2.550	0.015	0.093	0.008
AIL → FLCA → WTC	0.032	0.014	2.286	0.01	0.065	0.002
Total Mediation	0.096	0.024	4.000	0.053	0.147	0.000
Direct Mediation	0.060	0.044	1.363	-0.025	0.148	0.161
Total Effect	0.156	0.039	4.000	0.078	0.232	0.001

Note: SE—standard error; LL represents the lower limit of the bias-corrected 95% confidence interval, whereas UL indicates the upper limit of the bias-corrected 95% confidence interval.

## Data Availability

Data will be made available upon reasonable request.
